# Supplementary material for: Mineralization of 3D Osteogenic Model Based on Gelatin-Dextran Hybrid Hydrogel Scaffold Bioengineered with Mesenchymal Stromal Cells: A Multiparametric Evaluation
Source: Materials (Basel). 2021 Jul 9;14(14):3852. doi: 10.3390/ma14143852 (PMC8306641; doi:10.3390/ma14143852)
Supplement: Supplementary file 1 [file materials-14-03852-s001.zip › materials-1222596-supplementary.pdf]

materials

Supplementary Materials

Mineralization of 3D Osteogenic Model Based on Gelatin-Dextran Hybrid Hydrogel Scaffold Bioengineered with Mesenchymal Stromal Cells: A Multiparametric Evaluation

Federica Re [1,2], Luciana Sartore [3], Elisa Borsani [4], Matteo Ferroni [5,6], Camilla Baratto [7], Allia Mahajneh [8], Andrew Smith [8], Kamol Dey [3,9], Camillo Almici [10], Pierangelo Guizzi [11], Simona Bernardi [1,2], Guido Faglia [7,12], Fulvio Magni [8] and Domenico Russo [1,*]

[1] Bone Marrow Transplant Unit, Department of Clinical and Experimental Sciences, University of Brescia, ASST Spedali Civili, Piazzale Spedali Civili 1, 25123 Brescia, Italy; federicare91@gmail.com (F.R.); simona.bernardi@unibs.it (S.B.)
[2] Centro di Ricerca Emato-Oncologica AIL (CREA), ASST Spedali Civili, Piazzale Spedali Civili 1, 25123 Brescia, Italy
[3] Department of Mechanical and Industrial Engineering, University of Brescia, Via Branze 38, 25123 Brescia, Italy; luciana.sartore@unibs.it (L.S.); k.dey@unibs.it (K.D.)
[4] Division of Anatomy and Physiopathology, Department of Clinical and Experimental Sciences, University of Brescia, Viale Europa 11, 25123 Brescia, Italy; elisa.borsani@unibs.it
[5] Department of Civil, Environmental, Architectural Engineering and Mathematics (DICATAM), University of Brescia, Via Valotti 9, 25123 Brescia, Italy; matteo.ferroni@unibs.it
[6] CNR-IMM Bologna, Via Gobetti 101, 40129 Bologna, Italy
[7] PRISM Lab, CNR-INO, 25123 Brescia, Italy; camilla.baratto@unibs.it (C.B.); guido.faglia@unibs.it (G.F.)
[8] Clinical Proteomics and Metabolomics Unit, Department of Medicine and Surgery, University of Milano-Bicocca, Via Raoul Follereau 3, 20854 Vedano al Lambro, Italy; allia.mahajneh@unimib.it (A.M.); andrew.smith@unimib.it (A.S.); fulvio.magni@unimib.it (F.M.)
[9] Department of Applied Chemistry and Chemical Engineering, Faculty of Science, University of Chittagong, Chittagong 4331, Bangladesh
[10] Laboratory for Stem Cell Manipulation and Cryopreservation, Department of Transfusion Medicine, ASST Spedali Civili, Piazzale Spedali Civili 1, 25123 Brescia, Italy; camillo.almici@asst-spedalicivili.it
[11] Orthopedics and Traumatology Unit, ASST Spedali Civili, Via Papa Giovanni XXIII 4, 25063 Gardone Val Trompia, 25123 Brescia, Italy; pieroguizzi@tiscali.it
[12] Department of Information Engineering (DII), University of Brescia, Via Branze 38, 25123 Brescia, Italy
* Correspondence: domenico.russo@unibs.it

Table S1. Overview of the phospholipid species identified by in situ MALDI-MS/MS LID-LIFT™ fragmentation. Lipid identification was obtained by cross-referencing the product ion spectra with lipids listed in the METLIN, HMDB, and Lipid Maps databases.

Precursor m/z	m/z product ions	Lipid ID
885.57	601, 599, 581, 419, 303, 283, 241, 153, 97, 79	PI(18:0/20:4)
863.57	599, 581, 419, 283, 281, 241, 153, 97, 79	PI(18:0/18:1)

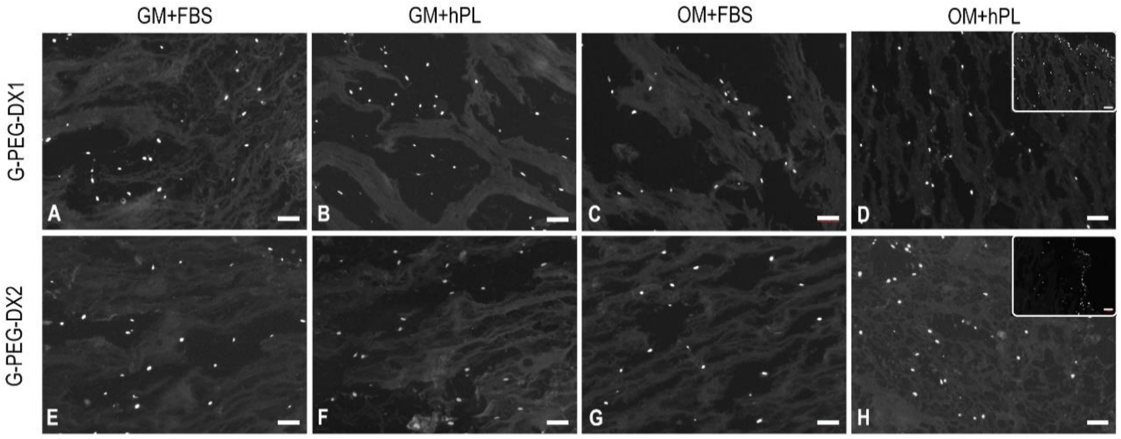

Figure S1. Microphotographs of DAPI positive cell nuclei for cell colonization. Microphotographs of G-PEG-Dx1 (**A–D**) and G-PEG-Dx2 (**E,H**) under the different culture conditions: GM+FBS (**A,E**), GM+hPL (**B,F**), OM+FBS (**C,G**), OM+hPL (D,H). Scale bar: 500 μm.

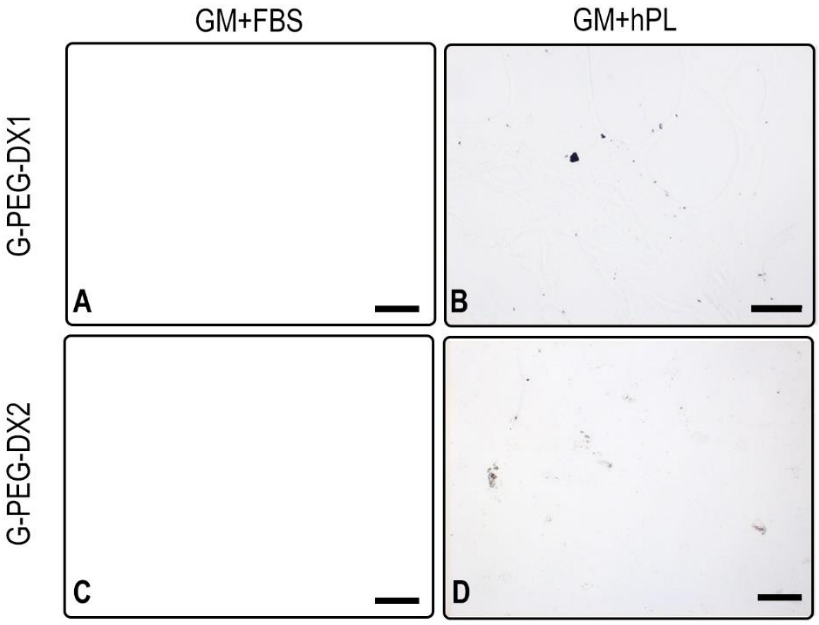

Figure S2. Calcium deposit distribution in hydrogels using Von Kossa stain. Microphotographs of G-PEG-Dx1 (**A,B**) and G-PEG-Dx2 (**C,D**) under GM-FBS (**A,C**) and GM-HPL (**B,D**). Scale bar (A,B,C) 100 μm and scale bar (B) 20 μm. A,C appears without deposits**.**
